# Automated evaluation of dental cavity preparation quality using deep learning and anatomically informed geometric analysis

**DOI:** 10.3389/frai.2026.1884843

**Published:** 2026-08-11

**Authors:** Abdullah F. Alshammari, Bassam A. Anazi, Mahvish Khan, Hamdan A. Alshammari, Najmah A. Almowina, Yousef E. Alenezi, Saif Khan, Shafiul Haque, Ahmed A. Madfa

**Affiliations:** 1 Department of Basic Dental and Medical Science, College of Dentistry, University of Ha’il, Ha’il, Saudi Arabia; 2 Department of Biology, College of Science, University of Ha’il, Ha'il, Saudi Arabia; 3 Dental Research Centre, College of Dentistry, University of Ha’il, Ha’il, Saudi Arabia; 4 Ministry of Health, Ha'il Health Cluster, Ha'il, Saudi Arabia; 5 Department of Nursing, College of Nursing and Health Sciences and Health Research Center, Jazan University, Jazan, Saudi Arabia; 6 School of Medicine, Universidad Espiritu Santo, Samborondonl, Ecuador; 7 Department of Restorative Dental Science, College of Dentistry, University of Ha’il, Ha’il, Saudi Arabia

**Keywords:** artificial intelligence, class I cavity preparation, deep learning, dental cavity preparation, image-based assessment

## Abstract

**Background:**

The quality of cavity preparation critically influences the longevity and success of restorative dental treatments. Current assessment methods remain largely subjective, relying on visual inspection and examiner judgment, which are prone to variability and limited reproducibility. Although three-dimensional (3D) imaging enables quantitative evaluation, its routine use in clinical and educational settings is limited by cost, accessibility, and workflow complexity.

**Objective:**

This study aimed to develop an automated, objective, and clinically interpretable framework for evaluating dental cavity preparation quality using standard two-dimensional (2D) images, with optional integration of 3D depth information.

**Methods:**

A deep learning pipeline based on enhanced U-Net architectures was developed to automatically segment cavity and cusp regions from 2D molar photographs. Anatomically informed geometric analyses were applied to quantify cavity-shape similarity, intercuspal distance, isthmus width, and cavity proportionality. Global cavity-shape conformity was assessed using Elliptic Fourier Descriptors (EFDs), enabling scale-, rotation-, and translation-invariant comparisons with reference preparations. When 3D STL data were available, cavity depth and cavity-bed smoothness were additionally quantified. These measurements were integrated into a transparent Cavity Quality Score (CQS) ranging from 1 to 10.

**Results:**

The cavity segmentation model achieved an internal validation Dice coefficient of 0.81 and an Intersection-over-Union of 0.74, while cusp segmentation achieved a Dice coefficient of 0.83. External validation using measurements from three independent experts demonstrated close agreement between automated predictions and expert consensus for EFD cavity-shape similarity (MAE = 1.32 percentage points; *r* = 0.981), pooled isthmus-width measurements (MAE = 0.03 mm; *r* = 0.995), pooled cusp-pair distances (MAE = 0.08 mm; *r* = 0.999), and cavity depth estimation (absolute error ≈ 0.01 mm).

**Conclusion:**

This study presents a hybrid, explainable artificial intelligence framework for objective assessment of dental cavity preparation using widely available 2D images. By integrating deep learning with anatomically informed geometric analysis, the proposed CQS offers a transparent and scalable tool for formative feedback in clinical and competency-based dental education. Further validation against expert summative grading is required before high-stakes implementation.

## 1 Introduction

Dental caries management and restorative dentistry fundamentally depend on the quality of cavity preparation, as cavity geometry, depth, isthmus width, and surface regularity directly influence restoration longevity, marginal adaptation, fracture resistance, and secondary caries development. Poor adhesive performance and early repair failure have often been associated with suboptimal cavity design, such as over-preparation, insufficient depth, severe isthmus constriction, or uneven cavity floors (Schwendicke et al., 2020; Qutieshat et al., 2024; Revilla-León and Özcan, 2019). Therefore, one of the most important factors in establishing the long-term efficacy of restorative treatments is the objective and regular evaluation of cavity preparation quality.

Despite its importance, cavity quality evaluation is mostly subjective in routine clinical practice and dental education. Visual inspection and tactile probing, which are influenced by clinician competence, are prone to considerable inter- and intra-operator variability in preclinical training environments where student performance is manually graded (Chen et al., 2024; Vitai et al., 2023). Examiner bias, inconsistency, and limited scalability undermine the reliability of conventional evaluation methods. As dental schools adopt competency-based curricula, the need for standardised, objective, and automated cavity evaluation technology is growing (Mallineni et al., 2024).

Cavity depth, volume, wall angulation, and surface smoothness may be quantitatively analysed in detail using three-dimensional (3D) imaging modalities (Esteva et al., 2019; Umer et al., 2024). Research on cavity morphology and restorative adaptation has extensively used these technologies. However, cost, equipment availability, and process complexity limit their regular use for cavity quality evaluation (Schwendicke et al., 2019). On the other hand, intraoral and two-dimensional (2D) photographic pictures are readily available, reasonably priced, and often obtained in both clinical and educational contexts. Robust techniques for obtaining clinically significant cavity-quality measurements from 2D images are still few, despite their widespread use.

Recent developments in deep learning and artificial intelligence (AI) have revolutionised the interpretation of dental images. Convolutional neural networks (CNNs) have demonstrated high accuracy in anatomical landmark recognition, caries diagnosis, restoration analysis, and tooth segmentation (Chaves et al., 2023; Aljulayfi et al., 2024; Bhattacharjee, 2022). For pixel-level segmentation of dental features, encoder–decoder architectures such as the U-Net have been particularly successful, enabling accurate delineation of cusps and cavities (Oliveira, 2025). However, without converting segmentation outputs into anatomically based, clinically interpretable quality criteria, most published research concentrates only on detection or segmentation accuracy (Gaboutchian et al., 2021).

Cavity preparation quality is intrinsically multifaceted, including surface smoothness, depth adequacy, isthmus geometry, intercuspal connections, and global shape conformity. The therapeutic and educational value of current digital techniques is limited, as they seldom incorporate these characteristics into a single evaluation framework (Serbanoiu et al., 2024). Furthermore, despite their prevalence in actual dental workflows, few systems are built to function well with only 2D images.

The present study addresses these limitations by proposing a comprehensive, image-driven framework for automated cavity quality assessment, culminating in the formulation of a Cavity Quality Score (CQS) ranging from 1 to 10. To extract cavity and cusp anatomy from typical 2D molar photos, the proposed pipeline uses deep learning-based segmentation followed by anatomically informed geometric analysis of proportional relationships, intercuspal distance, isthmus width, and cavity-shape similarity. Importantly, the framework is intended as an interpretable assessment and feedback model rather than an autonomous replacement for faculty or clinician judgment. It operates independently of 3D data, ensuring broad applicability, while allowing optional augmentation using depth and cavity-bed smoothness measurements derived from STL files when such data are available.

Therefore, the specific objectives of this study were: (1) to develop and evaluate deep learning models for automated segmentation of cavity and cusp regions from standard 2D photographs of Class I molar cavity preparations; (2) to derive automated, anatomically interpretable measurements of cavity-shape similarity, isthmus width, intercuspal distance, cavity depth, and cavity-bed smoothness; (3) to compare the automated component measurements with expert-consensus measurements from independent dental experts; and (4) to present the Cavity Quality Score (CQS) as a transparent composite framework for structured formative feedback.

## 2 Methods

### 2.1 Acquiring and annotating image datasets

Two-dimensional RGB images of plastic/typodont molar teeth with Class I occlusal cavity preparations were obtained from reference preparations and student preparations at the College of Dentistry, University of Ha’il. After screening, 250 unique original 2D RGB images of Class I occlusal cavity preparations were retained. The total working set contained 225 (training: 175, validation: 50) image instances/epoch (till best epoch 73rd; training: 175 × 73 = 12,775 and Validation: 50 × 73 = 3,650). Images were acquired using a standard tooth-imaging protocol: the molar tooth was positioned with the occlusal surface facing the camera, the tooth was centred in the field of view, the camera was oriented approximately perpendicular to the occlusal plane, and uniform illumination with a non-distracting background was used to minimize shadows, glare, and perspective distortion. Images were included if they showed a plastic molar tooth with a visible Class I cavity preparation, complete visualisation of the occlusal surface and relevant cusps, clearly visible cavity margins, adequate focus and exposure, and no identifiable personal information. Images were excluded if they were blurred, overexposed or underexposed, affected by marked glare or shadow, showed incomplete tooth/cavity boundaries, contained occluding objects or debris, had severe angulation or perspective distortion, represented non-Class I preparations, were duplicates, corrupted files, or lacked corresponding annotation files, some images freely available on internet in the public domain were also used.

In Computer Vision Annotation Tool (CVAT), separate segmentation assignments were created for the two target anatomical regions: the main cavity and the cusp regions. The main cavity mask included only the prepared occlusal cavity outline visible in the 2D image and excluded background, external tooth surface, shadows, scratches, and isolated artefacts. The cusp mask included the visible cusp regions required for intercuspal-distance analysis and excluded the cavity, background, and non-cusp tooth areas. Ground-truth masks were manually delineated by trained annotators (Two in this case) using polygon-based segmentation tools in CVAT and were subsequently checked for boundary completeness and label consistency before preprocessing. Ground-truth masks were manually delineated by two trained annotators and reviewed by a senior dental faculty member; disagreements were resolved by senior endodontist/s.

To validate the reliability of the Class I cavity annotations, a random subset of molar tooth images (15% of the total dataset) was independently segmented by both Annotator 1 and Annotator 2. Spatial consistency was evaluated using the Dice Similarity Coefficient (DSC). The mean inter-annotator score across the validation set was 0.84 ± 0.06, indicating high spatial consensus. Final boundaries for discrepant cases were established by a tie-breaking review by a senior endodontist.” To evaluate intra-annotator stability and guard against fatigue-induced drift, Annotator 1 re-annotated a blinded, shuffled subset of 15% images 3 weeks after their initial session. The intra-annotator DSC score was 0.89 ± 0.04, demonstrating excellent reproducibility in delineating Class I cavity margins over time.”

### 2.2 Standardisation and reproducibility of imaging data

All images were obtained as RGB photographs with varying spatial resolutions and orientations. To ensure that all images and their corresponding masks were consistently sized and suitable for training, they were uniformly rescaled to a fixed spatial resolution of 256 × 256 pixels with three colour channels. To avoid label interpolation anomalies, binary masks were processed using nearest-neighbour interpolation, whereas intensity images were resampled using appropriate interpolation methods. For reproducibility, random seeds for NumPy and TensorFlow were set prior to preprocessing.

### 2.3 Automated tooth identification and cropping

Raw photographs often include extensive background regions that are not directly relevant to cavity or cusp morphology, potentially complicating the analysis unnecessarily. To fix this, an automated localisation pipeline was established. The first step involved converting the images to black and white. Subsequently, the foreground was separated from the background, and the outer edges were trimmed. The largest connected component, corresponding to the tooth, was then identified and enclosed within a tightly fitted bounding box with minimal padding to preserve surrounding anatomical structures. During training, the cropped region was resized to the target resolution. This improved spatial accuracy while keeping background noise lower. This preprocessing method aligns with common methods used in biomedical and dental image segmentation (Ronneberger et al., 2015).

### 2.4 Enhancing masking and filtering architectures

Connected-component analysis was applied to refine the binary cavity masks and reduce annotation noise. Only the largest connected region, representing the main cavity structure, was retained. This step ensured that the model focused on anatomically relevant regions rather than small, potentially misleading artefacts that could disrupt learning. Similar filtering approaches are commonly employed in biomedical image segmentation workflows (Umbaugh, 2023).

### 2.5 PCA-based alignment for geometric normalisation

Geometric normalisation was applied to both the training and validation datasets to enhance sample uniformity. Principal component analysis (PCA) was performed on the foreground cavity pixels to determine the primary anatomical axis. This axis was then used to rotate the intensity images, cavity masks, and cusp masks to achieve a consistent vertical orientation. Cubic interpolation was applied to intensity images, whereas nearest-neighbour interpolation was used for binary masks to preserve label integrity. This PCA-based alignment reduces pose variability while maintaining anatomical orientation, consistent with common practices in medical image shape normalisation (Jolliffe and Cadima, 2016). All molar teeth obtained from varied sources were scaled to a uniform size (256 × 256, example in Supplementary Figure 1S) and vertically aligned to the cavity principal vertical axis. These vertically aligned images were subjected to on-the-fly data augmentation to enhance model generalisation and mitigate overfitting.

### 2.6 Uniform preprocessing of training and validation datasets

After image screening, 250 unique 2D RGB images of Class I molar cavity preparations were retained. These were divided at the image level into training, validation, and test/sample subsets using a 70:20:10 split, corresponding to 175 training images, 50 validation images, and 25 test/sample images. The total working set contained 225 (training: 175, validation: 50) image instances/epoch. Training images were used for model optimisation, validation images were used for internal model monitoring and model selection, and test/sample images were reserved for post-training inference and quantitative analysis.

All training and validation images underwent the same deterministic preprocessing pipeline, including tooth localisation, cropping, resizing to 256 × 256 pixels, mask refinement, and PCA-based vertical alignment. Binary cavity and cusp masks were resized using nearest-neighbour interpolation to preserve label boundaries, whereas RGB images were resized using image-intensity interpolation. Random data augmentation was applied only to the training set during model fitting; validation and test/sample images were not augmented. Because the split was performed at the image level rather than at the specimen or student level, this limitation has been acknowledged when interpreting internal validation performance and potential information-leakage risk.

### 2.7 Data augmentation

Keras sequence generators were used to generate augmented data on the fly during training, thereby enhancing the model’s generalisability and adaptability. Identical geometric modifications were applied simultaneously to both the photographs and their corresponding masks. For the validation set, however, all random augmentations were omitted and no changes were applied.

The selected augmentations were designed to mimic realistic variations around the cavity area: random rotations up to ±15°, translations within ±10%, scaling by ±10%, shearing up to ±15°, and both horizontal and vertical flips. Luminance was additionally adjusted to a range of 0.8–1.2, but only for the intensity images. The masks were subjected solely to geometric transformations and were resampled using nearest-neighbour interpolation to preserve sharp boundaries and avoid artefacts. The batch size was kept constant at 8 throughout training.

For the cavity segmentation task, RGB images were paired with their corresponding binary cavity masks. Prior to network input, image intensities were normalised to the range, following standard practice for segmentation models (Ronneberger et al., 2015).

To achieve clear class separation for cusp segmentation, the colour-coded multi-class masks were converted into single-channel label maps using a channel-dominance approach (i.e., selecting the strongest channel per pixel). Identical geometric augmentations were applied to both images and masks, and consistent background padding was maintained for the masks. In line with established guidelines (Goodfellow et al., 2016; Shorten and Khoshgoftaar, 2019), all photometric (colour or brightness) transformations were excluded from the validation data.

### 2.8 Model for cavity segmentation

An enhanced U-Net deep learning model was employed to learn cavity segmentation. For training, a batch size of 8 was used for up to 1,000 epochs, with model performance evaluated after each epoch on validation data. The highest-performing model was used to generate cavity masks for test data. Binary cavity masks were generated by thresholding predicted sigmoid probability maps. To justify the threshold choice, an internal threshold-sensitivity analysis was performed on the validation set using thresholds of 0.40, 0.45, 0.50, 0.55, and 0.60. The highest Dice coefficient and IoU were obtained at the 0.50 threshold; therefore, 0.50 was retained for final binary mask generation. This fixed threshold was then applied consistently to all test/sample images before downstream EFD-based cavity-shape analysis, isthmus-width measurement, and CQS calculation.

**Figure 1:**
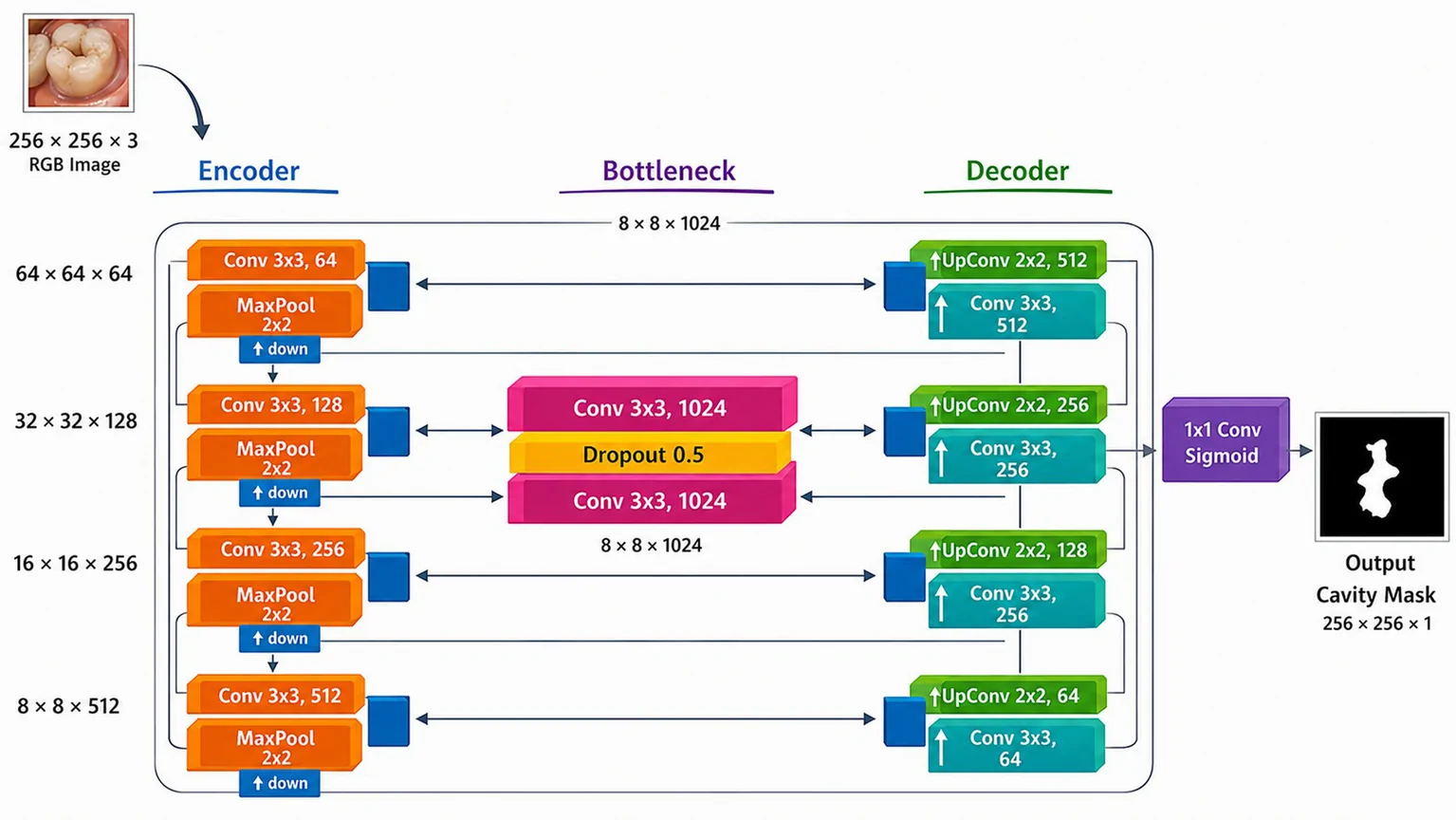
Refined encoder-decoder U-Net architecture for automated binary segmentation of Class I cavity preparations from standard two-dimensional molar photographs.

The final U-Net Model architecture consists of an encoder with four down-sampling stages, each comprising two consecutive 3 × 3 convolutional layers followed by batch normalisation and ReLU activation. Feature depths were progressively increased from 64 to 512 filters, allowing hierarchical extraction of low- to high-level representations. Spatial resolution was reduced using max-pooling layers after each encoder block (Figure 1).

At the network bottleneck, two 3 × 3 convolutional layers with 1,024 filters were employed, followed by batch normalisation and ReLU activation. A dropout layer (rate = 0.5) was introduced at this stage to reduce overfitting and improve generalisation.

The decoder mirrors the encoder structure, using transposed convolutions (2 × 2, stride = 2) for up-sampling. Skip connections concatenate corresponding encoder feature maps with decoder activations, enabling recovery of fine boundary details. Each decoder block contains two convolutional layers with batch normalisation and ReLU activation. The final output layer is a 1 × 1 convolution with sigmoid activation, producing a binary cavity probability map at the original image resolution (256 × 256 pixels). This architecture is conceptually aligned with the original U-Net design, which has proven highly effective for biomedical image segmentation tasks requiring precise boundary delineation (Ronneberger et al., 2015).

### 2.9 Loss function and optimisation

To robustly capture cavity morphology and boundary information, a composite loss function was employed, combining three complementary components:

Binary cross-entropy (BCE) – penalises pixel-wise misclassification.

Dice loss – maximises spatial overlap between predicted and reference masks, particularly effective for class-imbalanced segmentation problems (Equation 1).

Edge-aware loss – computed using Sobel gradient operators applied to both predicted and reference masks, explicitly enforcing boundary alignment.

The final loss function is defined as (Equation 1):


Loss=BCE+Dice Loss+0.5×Edge Loss (Eq. 1)

This formulation encourages both global region agreement and accurate cavity boundary detection, both of which are critical for dental morphology analysis. Similar hybrid loss strategies have been shown to improve segmentation accuracy in medical imaging applications (Sudre et al., 2017).

During training, two callbacks were employed:

Model checkpoint saves the model weights corresponding to the highest validation IoU score.

Early stopping: halts training if validation IoU does not improve for 20 consecutive epochs, with automatic restoration of the best-performing weights.

This strategy prevents overfitting while ensuring optimal model selection based on segmentation performance rather than training loss alone.

Model optimisation was performed using the Adam optimiser with a learning rate of 1 × 10^−4^, selected for its stability and fast convergence properties in deep segmentation networks (Kingma, 2014).

Model performance was quantitatively assessed using validation data:

Dice coefficient, measuring overlap between predicted and reference cavity regions.

IoU, evaluating segmentation accuracy at the pixel level.

### 2.10 Employing elliptic Fourier descriptors

Elliptic Fourier descriptors (EFDs) were employed to compare the shape and contours of the predicted cavity mask with the reference standard prepared cavity masks. Closed contours were generated for both the test and reference binary cavity masks. The closed contours were then used for extracting shortened Fourier descriptors. The Euclidean distances between Fourier descriptor vectors of predicted and reference masks were represented as percentage distances or similarity scores. This approach is free of any scale, rotation, or translation variation across the two matching binary cavity masks. EFD-based shape analysis is a well-established procedure in the study of biological and dental morphology (Falcão et al., 2017; Thapa et al., 2025).

### 2.11 Cusp segmentation and intercuspal distance

Cusp identification was performed via binary segmentation of cusp masks. Each mask was generated manually using CVAT as discussed earlier. Each binary mask (white foreground and black background) was resized to 256 × 256. Intensity thresholding was performed to strictly maintain the binary masks. The molar tooth images and their corresponding binary masks were stored in organised directories to facilitate on-the-fly data augmentation.

A deep U-Net Segmentation model, similar in design to the one used for cavity discovery (with minor modifications), was employed for cusp discovery (details in the results section). This model utilises mixed-precision arithmetic and the AdamW optimiser, with gradient clipping. The validation set was used to assess the model performance after each epoch (epochs: 1,000, batch size: 8). The highest foreground intersection-over-union (IoU) was used as the selection criterion for the best model, along with early stopping and learning rate scheduling.

### 2.12 U-net model for cusp region discovery

**Figure 2:**
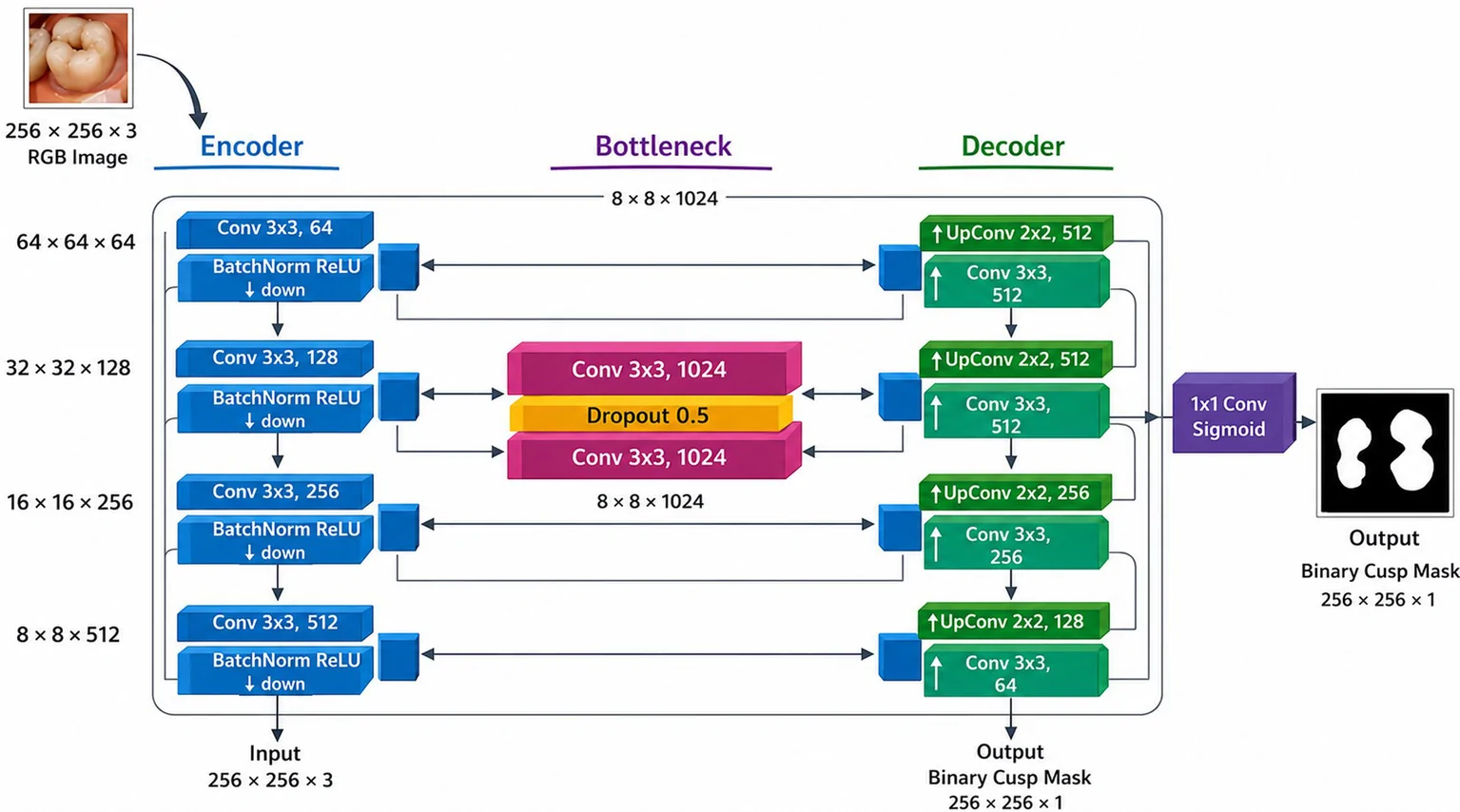
Refined U-Net-based cusp-region segmentation architecture for automated identification of anatomical cusp landmarks used in intercuspal-distance measurement.

The U-Net model for cusp region discovery comprises an encoder with five hierarchical levels, each composed of a three-convolution block with 3 × 3 kernels, batch normalisation, and ReLU activation (Figure 2). Compared with the classical two-convolution U-Net, the additional convolution per block increases representational capacity and improves sensitivity to subtle cusp morphology.

The number of feature channels increases progressively across encoder levels:

64 → 128 → 256 → 512 → 512 filters

Spatial down-sampling is achieved using 2 × 2 max-pooling layers. Dropout regularisation is selectively applied at deeper encoder levels (rates of 0.1 and 0.2) to reduce overfitting.

At the bottleneck, a convolutional block with 1,024 filters and a dropout rate of 0.3 captures high-level contextual information relevant to cusp anatomy.

Decoder and output layer: The decoder mirrors the encoder structure, using 2 × 2 transposed convolutions for up-sampling, followed by concatenation with corresponding encoder feature maps through skip connections. Each decoder stage applies a three-convolution block to refine spatial detail after feature fusion.

The output layer consists of a 1 × 1 convolution producing two logits per pixel corresponding to background and cusp classes. No activation function is applied at this stage; softmax normalisation is used during loss computation and inference. This design follows best practices for numerically stable training under mixed-precision arithmetic (Ronneberger et al., 2015).

#### 2.12.1 Loss function and evaluation metrics

To address the pronounced class imbalance between background and cusp pixels and to improve boundary delineation, a composite loss function was employed, combining:

Weighted sparse categorical cross-entropy, prioritising cusp pixels.

Weighted Dice loss, optimising spatial overlap between predicted and reference cusp regions.

The final loss was defined as (Equation 2):


$$L_{\mathrm{oss}}=0.5\times L_{\mathrm{wce}}+0.5\times L_{\text{Dice}}$$ (Eq. 2)

This hybrid formulation balances pixel-level accuracy with region-level overlap, a strategy widely adopted in medical image segmentation tasks with class imbalance (Sudre et al., 2017).

AdamW was selected for model optimisation due to its decoupled weight-decay formulation, which improves generalisation in deep convolutional models (Loshchilov and Hutter, 2017). A batch size of 8 was used, and training was conducted for up to 500 epochs, subject to early stopping. Model performance was evaluated using foreground-only metrics, excluding background pixels to avoid artificially inflated scores:

Foreground IoU.

Foreground Dice coefficient.

These metrics provide a clinically meaningful assessment of cusp segmentation accuracy.

Morphological techniques were used to enhance the predicted cusp masks and to create a careful, ‘strict’ foreground mask, in line with standard shape-analysis methods (Lotufo et al., 2023). Distance-transform-guided watershed segmentation was employed to delineate individual cusps clearly. This is a well-established approach for segmenting contacting anatomical structures (Areeckal et al., 2018).

The cusp centres were identified as the centres of the largest inscribed circles. The inscribed-circle approach follows strict boundary and distance constraints to avoid narrow extensions, sutures, and segmentation artefacts. A minimum threshold distance of approximately 3 mm was kept between the two cusps on the same side of the cavity. This value was used as a practical anatomical safeguard for typodont molar images rather than as a universal biological constant; future calibration should adjust this value by tooth type, image scale, and institutional assessment rubric.

This description, based on the medial axis, provides a robust, shape-consistent way to indicate the cusp’s location (Falcão et. al 2017).

The buccal and lingual sides of the cusp were demarcated by the central vertical axis passing through the centroid. Intercuspal pairs were then defined based on an angular criterion allowing a maximum transverse deviation of 70 degrees from the central axis. The angle threshold was selected to prevent anatomically implausible cross-pairing while preserving tolerance for modest image rotation and cusp-mask asymmetry. These geometric constraints are therefore intended as reproducible rule-based safeguards and should be evaluated in future sensitivity analyses.

### 2.13 Justification of geometric constraints

The geometric thresholds used for cusp pairing and isthmus detection were introduced to reduce failure modes that commonly occur when binary masks contain small spurs, narrow bridges, or partial cusp labels. Minimum centre-to-centre distances, angular limits, terminal buffers, and minimum-width filters therefore function as deterministic safeguards that make the algorithm reproducible and inspectable. They should not be interpreted as universal biological constants. Their final values should be confirmed by sensitivity analysis and by agreement with expert dental assessors.

### 2.14 Isthmus discovery and width

Isthmus discovery was performed on predicted binary cavity masks as discussed above (details in the results section). PCA was performed to determine the longest vertical axis passing through the cavity foreground. Boundary buffers were employed to avoid any narrow extensions or sutures in the predicted masks. Terminal boundary constraints were used to avoid isthmus discovery near boundary vertical extremities.

A medial axis (skeleton) was extracted, and the local cavity width was assigned to be twice the distance-to-boundary value at each skeleton point, in accordance with established local-thickness formulations (Dahl and Dahl, 2023; Miettinen et al., 2025). The application of orientation-based criteria for removing lateral branches refined skeleton graphs, thereby leading to more stable measurements (Li et al., 2025). The central line was determined using shortest-path discovery (Mnih et al., 2015).

### 2.15 Cavity depth estimation

Normalised 3D Computer-aided Design and Computer-aided Manufacturing (CAD/CAM) files were used to determine cavity depth when 3D STL data were available (Eggmann and Blatz, 2024). The predicted 2D binary cavity mask was utilised to identify the location and shape of the cavity within the 3D mesh. Depth values were interpreted only when image-to-mesh scaling information was available, because uncalibrated STL or photographic coordinate systems can introduce systematic dimensional error. For each STL file, the mesh was normalized and cropped to the central tooth region. The predicted two-dimensional cavity mask was registered to the STL mesh by mapping the mesh X-Y coordinates to the corresponding pixel coordinates of the binary cavity mask. Vertices projecting within the foreground cavity mask were retained as the initial cavity region and refined using the local depth field. When mask-based projection was insufficient, a depth-connectivity fallback was used to identify the central cavity component. Depth was calculated relative to a reference geometric surface, with the sign oriented so that the cavity floor corresponded to the deeper region. Scaled depth was calculated as $D_{i}=s(Z_{\mathrm{ref}}-z_{i}).$

where $Z_{\mathrm{ref}}$ was the reference height within the cavity box and “s” was the calibrated STL-to-millimeter conversion factor. The final cavity-depth value was calculated as the mean of the maximum depths across three equal cavity-bed regions, while depth smoothness was calculated as the standard deviation of these three regional maximum values.

### 2.16 External expert validation of automated component measurements

A standard expert-validation design was used to compare automated component measurements with independent expert assessment. From the 250 unique original images, 10% were selected for expert validation (25 for efd similarity, 24 for cusp distances and 20/isthmus for isthmus width). These cases were selected after image screening and were not treated as augmented samples. Three independent dental experts evaluated the same cases. For each measurable output, the mean of Expert 1, Expert 2, and Expert 3 was treated as the expert-consensus reference.

Automated predictions were compared with expert consensus using mean bias, mean absolute error (MAE), mean absolute percentage error (MAPE), and Pearson correlation. Sample size was reported separately for each validation domain because the number of paired observations differed across EFD similarity, pooled isthmus-width measurements, pooled cusp-pair-distance measurements, and cavity-depth analyses. For pooled isthmus-width and cusp-pair-distance analyses, two measurements were available per expert-validated case; therefore, 20 expert-validated cases yielded up to 40 paired measurements. Pearson correlation was reported only when at least three paired observations were available. Confidence intervals for Pearson correlations were calculated using Fisher z-transformation.

Inter-rater reliability among the three experts was assessed before averaging expert measurements into a consensus reference. A two-way random-effects, absolute-agreement intraclass correlation coefficient for average measures, ICC(A,3), was used because the expert-consensus value was defined as the mean of three independent expert ratings. Ninety-five percent confidence intervals were estimated using bootstrap resampling of measurement targets.

## 3 Results

### 3.1 U-net model for cavity identification

**Figure 3:**
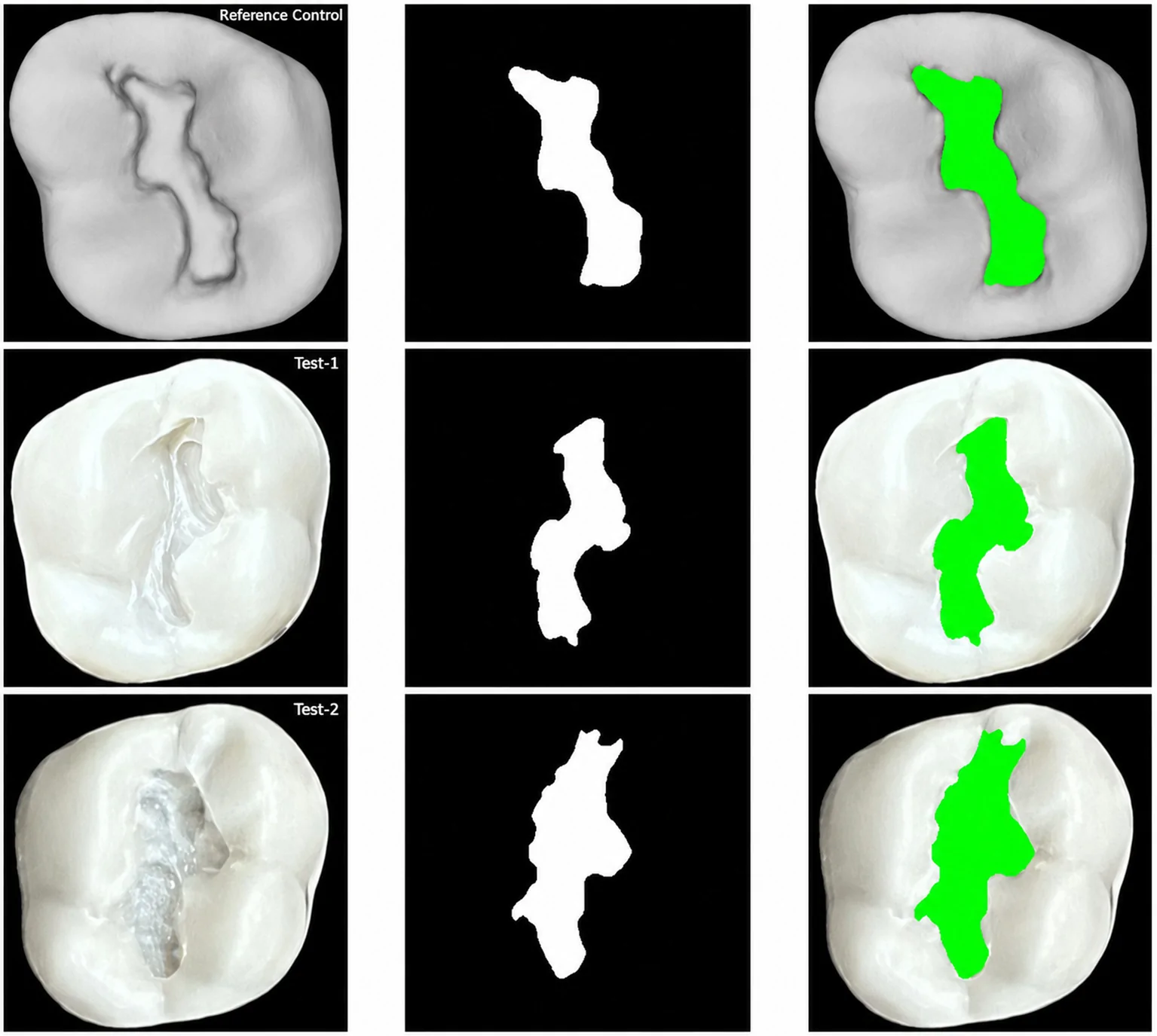
Representative automated Class I cavity segmentation output generated by the refined U-Net model from a standard two-dimensional molar photograph.

**Figure 4:**
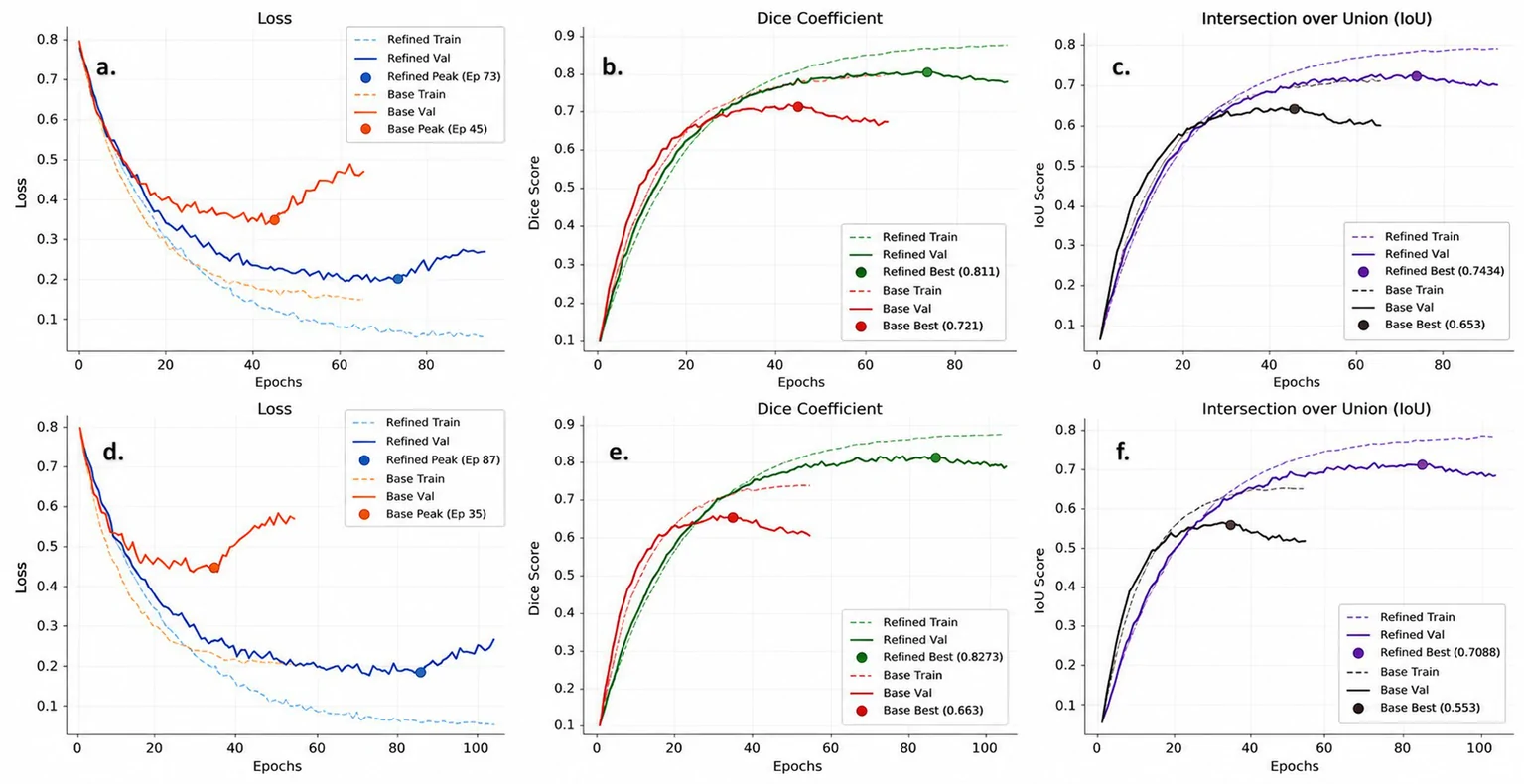
Training dynamics and baseline comparison of refined versus base U-Net models; (A–C) for Class I cavity and (d–f) Cusp Region segmentation using validation loss, Dice coefficient, and intersection-over-union.

The best-selected U-Net model for cavity prediction achieved an internal validation Dice coefficient of 0.811 and an IoU score of 0.7434, indicating good overlap between predicted and annotated cavity masks (Figure 3) for the available validation data (Figure 2; Figure 4). These metrics support technical feasibility for segmentation. Control refers to the standard cavity preparation of a reference molar tooth, and test refers to any test cavity preparation by a student or dentist. Learning-curve analysis compared the refined U-Net used in the final pipeline with a base U-Net model. The refined U-Net achieved higher validation performance than the base U-Net, with a Dice coefficient of 0.811 compared with 0.721 and an IoU of 0.7434 compared with 0.653. The best refined U-Net checkpoint was selected at epoch 73 based on validation performance, whereas the base U-Net reached its best validation performance at epoch 45. These results indicate that the refined U-Net improved segmentation performance and delayed overfitting compared with the base architecture (Figures 4a–c).

**Figure 5:**
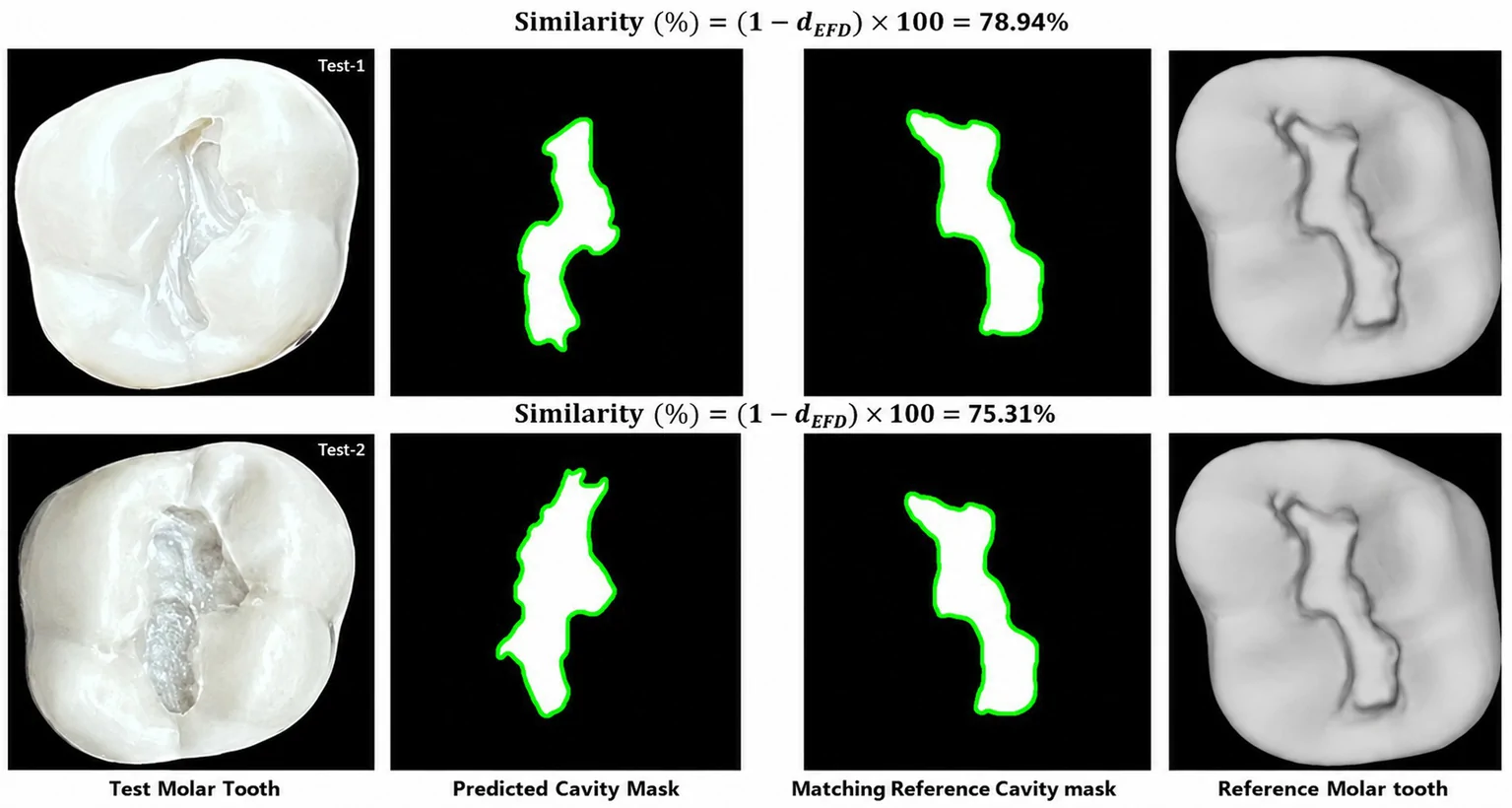
Elliptic Fourier Descriptor-based cavity-shape matching between the predicted cavity mask and the closest reference preparation.

The similarity scores were calculated using EFDs as discussed in the methods section. All the EFD similarity scores are saved in tabular form (Supplementary material: efd_similarity_scores.csv). The Algorithm searches for the closest match of the predicted cavity mask among the preprocessed reference cavity masks obtained from CVAT (Figure 5).

### 3.2 U-net model for cusp region discovery

**Figure 6:**
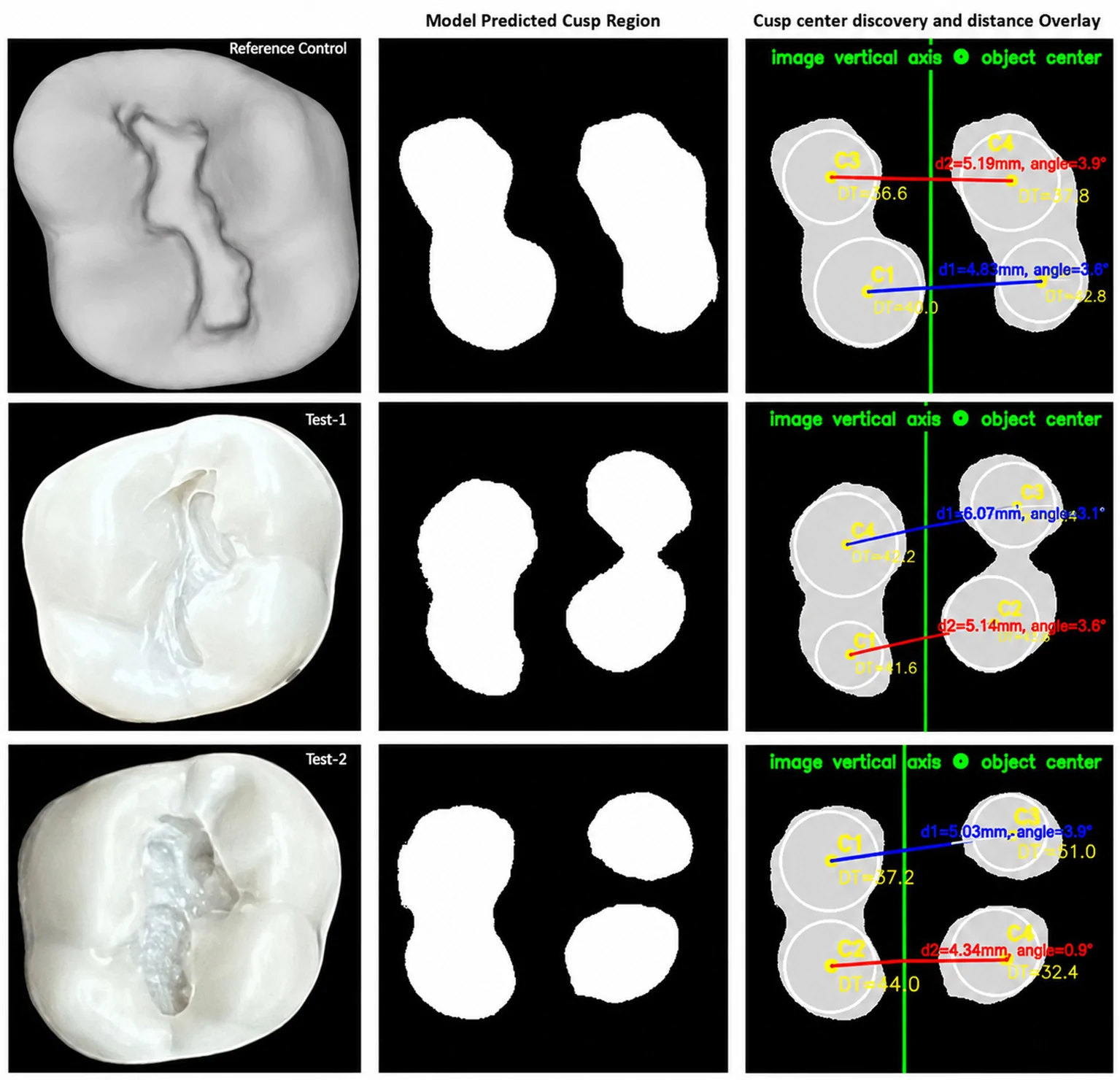
Automated cusp-region detection and intercuspal-distance measurement from refined U-Net cusp segmentation outputs.

The best-selected cusp model achieved a validation foreground IoU of 0.7088 and a foreground Dice coefficient of 0.8273, supporting good internal segmentation performance for the available cusp masks (Figure 6). This model was optimized to identify buccal-to-lingual cusp-tip relationships and did not yet detect all possible cusp-pair configurations, including mesiobuccal-to-mesiolingual relationships; this is now explicitly treated as a limitation and a priority for future model development. Learning-curve analysis compared the refined U-Net for Cusp region used in the final pipeline with a base U-Net model. The refined U-Net achieved higher validation performance than the base U-Net, with a Dice coefficient of 0.8273 compared with 0.663 and an IoU of 0.7088 compared with 0.553. The best refined U-Net checkpoint was selected at epoch 87 based on validation performance, whereas the base U-Net reached its best validation performance at epoch 35. These results indicate that the refined U-Net improved segmentation performance and delayed overfitting compared with the base architecture (Figures 4d–f).

### 3.3 Intercuspal distance calculation

For each selected cusp pair (as described in the methods section), the intercuspal distance was computed as the Euclidean distance between cusp centre coordinates (Equation 3):


$$d_{\mathrm{ij}}=\sqrt{{\left(x_{i}-x_{j}\right)}^{2}+{\left(y_{i}-y_{j}\right)}^{2}}$$ (Eq. 3)

Distances were initially expressed in pixel units. When spatial calibration information was available, pixel distances could be converted to physical units (e.g., millimetres) using a known pixel-to-length scaling factor. Euclidean distance between robustly defined cusp centres provides a reproducible and anatomically meaningful measure of intercuspal separation (Thapa et al., 2025). Visual overlays were generated showing cusp masks, estimated cusp centres, inscribed circles, the anatomical vertical axis, and the selected intercuspal connections (Figure 6). Quantitative results, including the number of cusps detected, intercuspal distances, and pairing angles, are summarised in a tabular format for downstream statistical analysis (cusp_pair_distances.csv available as Supplementary material).

### 3.4 Candidate isthmus and width

Along the ordered skeleton path (as discussed in the methods), a candidate set of positions was formed after applying three constraints:

Terminal exclusion (hard buffer): only skeleton points whose PCA projection t lies within [t_min + b_, t_max − b_], where b is the effective terminal buffer in pixels.

End trimming along the path: to further down-weight endpoint artefacts, a path margin of 10% at each end was removed (unless a high relaxation level was invoked).

Minimum ‘depth’ (radius) criterion: candidates were required to exceed a minimum medial-axis radius threshold (default 2 px, with a relaxation ladder described below).

Additionally, a central ‘band’ constraint was available to restrict candidates away from superior/inferior extremes: a PCA-based band using 15% quantile cutoffs (falling back to a row-band defined by bb_margin_frac = 0.15) could be enforced at the strictest level.

For each valid candidate i, the local width wi was computed as 2di and converted to millimetres using the calibration. The primary isthmus (Isthmus-1) was defined as the narrowest valid location along the constrained main axis. To avoid reporting clinically implausible micro-widths or segmentation noise, candidate widths were rejected if they were <0.5 mm, and the algorithm continued searching for the smallest width that met the minimum width requirement. The 0.5 mm rule was used as an image-analysis exclusion threshold for artefactual skeleton branches rather than as a definitive clinical acceptability threshold. If all valid candidates were below 0.5 mm, the method returned the widest available valid candidate as a fail-safe to avoid null outputs.

To locate a second, distinct constriction, the cavity was divided into two halves along the PCA axis using the median of foreground projections tmid. Isthmus-2 was then searched on the hard opposite half relative to Isthmus-1. To prevent selecting the same local constriction twice, a minimum centre-to-centre separation constraint of 2.5 mm was imposed and converted to pixels for the exclusion radius. This separation value was selected as a conservative spatial rule for typodont molar preparations and should be adjusted when the framework is applied to other tooth types, preparation designs, or camera scales.

If no valid Isthmus-2 candidate was found under strict constraints, a structured relaxation ladder was applied: (i) relaxing the central band requirement, (ii) lowering the minimum depth (radius) requirement, (iii) permitting broader path inclusion, and finally (iv) disabling pruning as a last resort while maintaining the opposite-half rule. This staged relaxation preserves anatomical intent (a distinct opposite-half constriction) while improving robustness against skeleton fragmentation and minor segmentation artefacts.

**Figure 7:**
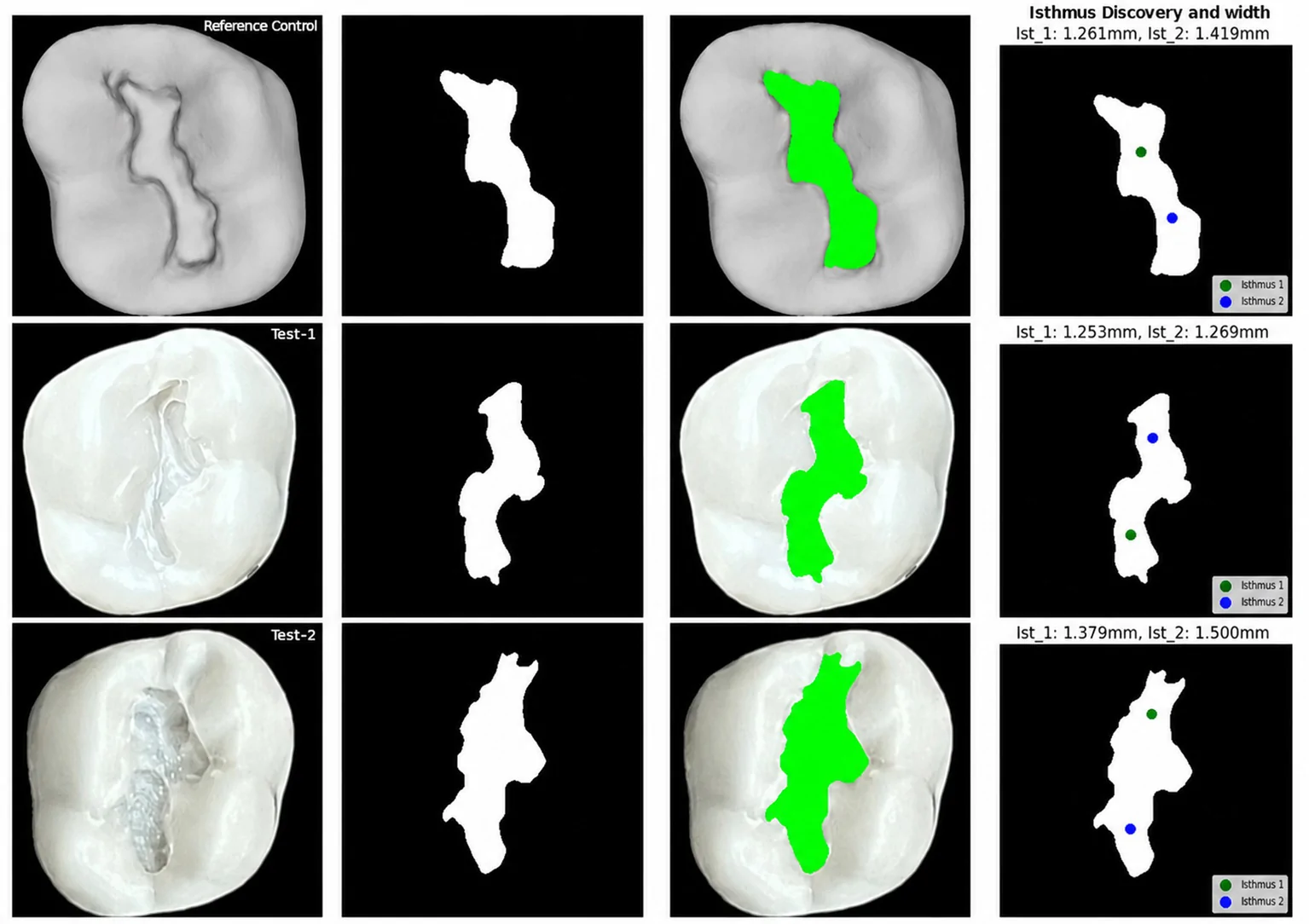
Skeleton-based automated isthmus-width detection from predicted Class I cavity masks.

For each mask, the method reported Isthmus-1 and Isthmus-2 widths in millimetres. Results were saved in a tabular output (isthmus_widths_two.csv available as Supplementary material), and overlaid visualisations were produced, displaying (i) the cavity region, (ii) the extracted skeleton, and (iii) the two detected isthmus centres (Figure 7).

### 3.5 Computation of the depth field on the cavity bed

A continuous depth field was defined on the 3D mesh derived from the STL files to describe vertical deviations associated with cavity formation. Depth was computed relative to a reference geometric surface using the best-fit plane. A reference plane was estimated from the vertex cloud using a least-squares formulation. The signed depth at each vertex was then calculated as the orthogonal distance from the vertex to this plane. This approach is commonly used in surface metrology to quantify local depressions and protrusions on scanned geometries (Li et al., 2020). The sign of the depth field was automatically normalised so that cavity regions corresponded to negative values, ensuring consistent interpretation across specimens. Cavity-bed depth-field analysis was completed for 50 STL files. For each case, the registered cavity region was used to generate a cavity-depth field, from which the regional maximum depths, mean cavity depth, and depth smoothness were calculated.

### 3.6 Projection of 3D geometry to 2D mask space

The predicted binary cavity mask from the trained U-Net segmentation model was projected onto the normalised 3D mesh (obtained 3D 3D CAD/CAM STL files). The 3D vertices falling within the 2D foreground of the binary cavity were retained as probable cavity regions within the 3D mesh.

The selected cavity region was then filtered to extract the central cavity, avoiding shallow or peripheral/boundary regions. To implement this, a minimum depth window was defined such that di ≤ dmin + *Δ*. Only those vertices with a minimum depth (dmin) were retained, and Δ is the depth tolerance.

Finally, the filtered central cavity region was then converted to a connected regional component containing the deepest vertices. This is a well-known standard approach of extracting anatomically similar regions in biological image data (Areeckal et al., 2018). A failsafe mechanism has also been incorporated in case the 2D binary mask fails to identify the cavity region; the algorithm then reverts to a purely depth-based connectivity strategy, thereby ensuring robustness in handling complex cavity shapes.

The filtered central cavity region was then enclosed in an axis-aligned cavity bounding box. This box was defined based on the range of the x, y, and z coordinates. The bounding box encloses a fractional margin percentage of the adjoining anatomy of the central cavity; this ensures that it avoids any unrelated anatomy while safely encapsulating the central cavity. The enclosed region, termed the cavity box, is the final 3D mesh for depth quantification.

The maximum value of the vertical coordinate (*z*) was used as a reference to determine the depth of the vertices within the cavity box, that is, the reference height was defined as:

Zref = max (z) within the cavity box

Scaled cavity depth at each vertex was then computed as: Di = s (Zref−zi)

where s is a scale factor converting z coordinate units to calibrated depth in pixels, followed by its conversion to mm. This assigns an inverse height scale to *z*-coordinate values. Max *z* values correspond to zero depth, while inversely increasing depth with decreasing *z* values. This is consistent with conventional relative-height analyses used in surface defect quantification.

Cavity datasets were generated as CSV files (cavity-only vertices, restricted to the central cavity component, and cavity-box vertices, including all vertices within the expanded bounding box) and are available as Supplementary material. The final cavity depth was represented as an average of the maximum depths along the three equal sections across the longest axis of the cavity. Then, the final cavity depth is given by the following equation (Equation 4):


$$D_{\text{cavity}}=\frac{1}{3}\sum_{k=1}^{3}\max{\left(D\right)}_{k}$$ (Eq. 4)

**Figure 8:**
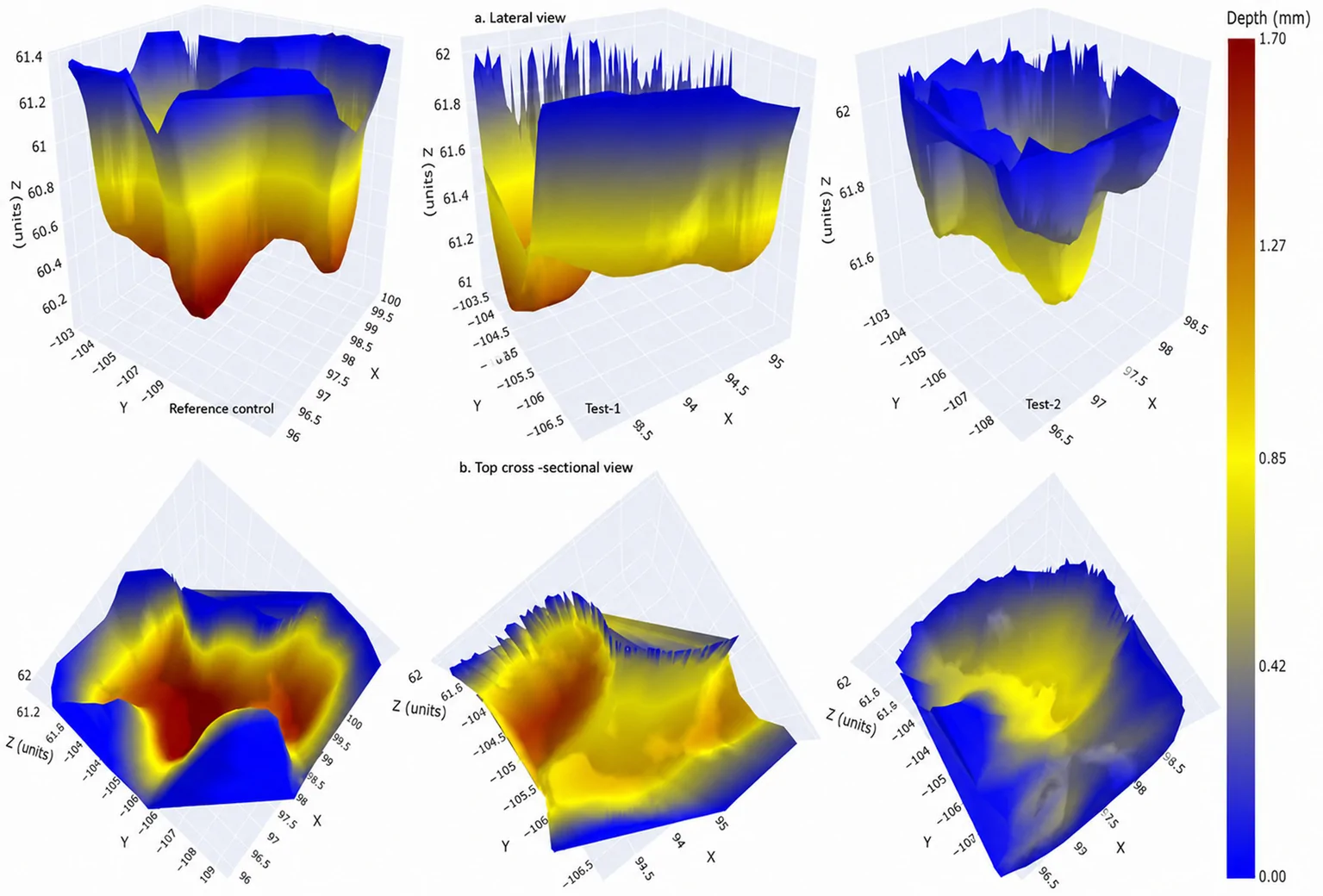
Three-dimensional STL-derived cavity-bed depth-field estimation using projected two-dimensional cavity masks and calibrated surface-depth analysis: **(A)** Lateral view; **(B)** top cross-sectional view.

The cavity depth obtained from the above equation avoids bias from extreme outliers and represents the depth of the overall central cavity, not just a specific region. Smoothness of the cavity bed was determined as a standard deviation of the maximum depths of the three regions along the longest axis of the central cavity. The depth and smoothness data are also provided in a separate CSV file (*_cavity_depth_summary.csv, available as* Supplementary material)*. The cavity box* (Figure 8) *is also provided as 3D interactive HTML files* (_cavity_box_3d_interactive.html), available as Supplementary material.

### 3.7 CQS: rationale for CQS weighting, thresholds, and penalty functions

The CQS was designed as an interpretable rule-based score rather than a purely data-fitted prediction model. The largest weight was assigned to EFD shape similarity (50%) because global outline conformity summarizes the overall cavity design and is available from 2D photographs. Depth and proportional isthmus/intercuspal geometry were assigned intermediate weights (20% each) because they represent clinically important preparation dimensions but depend on either 3D availability or scale calibration. Bed smoothness was assigned a lower weight (10%) because it is clinically relevant yet more sensitive to STL quality, scanner noise, and registration error. These weights are transparent starting values and should be re-estimated in future validation studies using expert-panel scores and outcome-linked calibration (Qutieshat et al., 2024; Ritter et al., 2019; Tejani et al., 2024; Collins et al., 2024).

Let:

SEFD = EFD cavity shape similarity (%)

D = cavity depth (mm), target range [1.5, 2.0 mm]

R = isthmus width/intercuspal distance (dimensionless), target R₀ = 1/3

*σ* = cavity bed smoothness = SD of the 3 regional maxima (mm), ideal 0

Four sub-scores normalized to [0,1] are computed and subsequently weighted:


$$\mathrm{CQS}=10(0.5Q_{\mathrm{EFD}}+0.2Q_{\text{Depth}}+0.2Q_{I/C}+0.1Q_{\text{Smooth}})$$ (5)

$$\mathrm{CQS}=10(0.7Q_{\mathrm{EFD}}+0.3Q_{I/C})$$ (5^´^)

Equation 5 is the scoring with an optional depth score, while Equation 5^´^ is the scoring without a depth score (in case 3D STL files are not available).

EFD shape similarity sub-score (50% weightage) is directly proportional to similarity percentage (Equation 6):


$$Q_{\mathrm{EFD}}=\frac{S_{\mathrm{EFD}}}{100}$$ (Eq. 6)

Depth sub-score (20% weightage) is penalised when the measured depth falls outside the target range of 1.5–2.0 mm. A soft linear penalty outside the accepted band is applied, with no penalty inside the band and a tolerance scale delta_D defining the distance at which the depth sub-score reaches zero (Equation 7). The target range was selected because it reflects commonly taught preclinical operative-dentistry criteria for conservative occlusal preparations in typodont training; however, the appropriate depth band may vary by material, lesion extent, tooth anatomy, and institutional rubric (Ritter et al., 2019).

Hence, the distance outside the depth range is (Equation 8):


$$\Delta_{D}=\{\begin{array}{c} 1.5-D,\;\;D<1.5\\ 0,2.0\;\;\;1.5\leq D\leq 2.0\\ D-2.0,\;\;\;D>2.0\; \end{array}\}$$ (Eq. 7)

$$Q_{\text{Depth}}=\max(0,1-\frac{\Delta_{D}}{t_{D}})$$ (Eq. 8)

Ratio sub-score (20% weightage) is penalised when the isthmus-width/intercuspal-distance ratio deviates from the target R0 = 1/3. A log-symmetric penalty was employed so that being proportionally too wide or too narrow is penalised symmetrically. A tolerance multiplier tau_R was used, with a practical default value of 1.5; this means that ratios within approximately +/−50% of the target incur only mild penalty, whereas larger deviations incur progressively stronger penalties. The mean intercuspal distance and mean isthmus distance are used for this ratio (Equation 9). This threshold reflects a conservative preclinical reference rather than a universal clinical rule, and should be calibrated against expert assessment before summative use (Qutieshat et al., 2024; Ritter et al., 2019).


QI/C=max(0,1−∣ln(RR0)∣ln(kr)) (Eq. 9)

Interpretation:


$$\text{if}\;R=R_{0}\to \ln(1)=0\to Q_{I/C}=1$$

ifR=1.5R0orR=R01.5→QI/C=0

Smoothness sub-score (10% weightage) applies a weak penalty for higher cavity-bed depth variability (sigma), where sigma is the standard deviation of the maximum depths of three regions along the longest axis of the central cavity. Because floor smoothness is clinically relevant but can be sensitive to mesh quality, scanner noise, and segmentation-to-STL registration error, it was assigned a lower weight than global outline, depth, and proportional geometry (Equation 10):


$$Q_{\text{Smooth}}=e^{\left(-\frac{\sigma }{t_{\sigma }}\right)}$$ (Eq. 10)

Final CQS:


$$\mathrm{CQS}=10(0.5\frac{S_{\mathrm{EFD}}}{100}+0.2Q_{\text{Depth}}+0.2Q_{I/C}+0.1e^{\left(-\frac{\sigma }{t_{\sigma }}\right)})$$ (11)

$$\mathrm{CQS}=10(0.7\frac{S_{\mathrm{EFD}}}{100}++0.3Q_{I/C})$$ (11^´^)

Equation 11 is the final scoring equation with optional depth score, while Equation 11^´^ is the final scoring equation without the depth score when 3D STL files are unavailable. The CQS values for the two available test cases remain unchanged: CQSTEST-1 = 6.25 (q_efd = 0.7894, q_depth = 0.844, q_ratio = 0.0, q_smooth = 0.6167) and CQSTEST-2 = 6.06 (q_efd = 0.7531, q_depth = 0.0, q_ratio = 0.782, q_smooth = 0.731). These examples demonstrate score generation and feature-level interpretability, but should not be interpreted as proof of expert-level grading validity without comparison to calibrated faculty ratings.

### 3.8 External expert validation of automated component measurements

The Supplementary material contain external expert validation of the automated predictions for EFD similarity, isthmus widths, cusp-pair distances, and cavity depth. Automated outputs were compared with the mean of three external experts. Overall, the automated measurements showed close agreement with expert consensus across all evaluated component outputs (Table 1).

**Table 1:** External expert validation of automated component measurements.

Validation domain	Supported data CSV	Number of expert validated samples	Automated prediction, mean +/− SD	Expert consensus, mean +/− SD	Bias	MAE	MAPE	Pearson *r*	95% CI for *r*
EFD cavity-shape similarity (%)	efd_similarity_scores (Supplementary material: efd_similarity_scores.csv)	25	84.61 +/− 7.89%	84.93 +/− 8.18%	−0.32%	1.32%	1.6%	0.981	0.96–0.99
Isthmus width (mm; isthmus 1 and 2 pooled)	isthmus_widths (Supplementary material: isthmus_widths_two.csv)	40	1.49 +/− 0.32 mm	1.49 +/− 0.33 mm	−0.00 mm	0.03 mm	2.0%	0.995	0.99–0.99
Cusp-pair distance (mm; pair 1 and 2 pooled)	cusp_pair_distances (Supplementary material: cusp_pair_distances.csv)	24	4.13 +/− 1.79 mm	4.13 +/− 1.78 mm	−0.00 mm	0.08 mm	1.8%	0.999	0.99–0.99
Test-1 cavity depth D (mm)	Test cavity depth (Supplementary material: Test-1_cavity_depth_summary.csv)	1	1.54 mm	1.53 mm	0.01 mm	0.01 mm	0.3%	NA	NA
Test-2 cavity depth D (mm)	Test cavity depth (Supplementary material: Test-2_cavity_depth_summary.csv)	1	0.85 mm	0.87 mm	−0.01 mm	0.01 mm	1.7%	NA	NA

MAE, mean absolute error; MAPE, mean absolute percentage error; SD, standard deviation; NA, not applicable because only one paired depth value was available for each test case. Bias is calculated as automated prediction minus expert consensus.

The final Cavity Quality Score (CQS) was validated against assessments from three independent dental experts. For each student-prepared cavity case, the AI-generated CQS was compared with the mean expert-consensus score derived from Expert 1, Expert 2, and Expert 3. The automated CQS values were within ±6% of the expert-consensus scores, indicating close agreement between the proposed composite scoring framework and expert summative evaluation.

Inter-rater reliability among the three experts was high across the evaluated domains (refer to ICC table in Supplementary material). The average-measures ICC(A,3) was 0.947 for EFD reference-match percentage, 0.989 for pooled isthmus-width measurements, 0.994 for pooled cusp-pair-distance measurements, and 0.995 for pooled regional depth maxima. These findings support the use of the mean expert score as the expert-consensus reference.

## 4 Discussion

This study provides a comprehensive and flexible framework for objective assessment of cavity preparation quality by combining deep learning-based photographic segmentation with anatomically grounded geometric and morphometric analysis. By combining modern deep learning architectures with conventional image-processing principles, the approach aligns with established foundations in computer vision and medical image analysis (Umbaugh, 2023; Goodfellow et al., 2016). The framework is supported by internal segmentation validation and by external expert validation of the automated component measurements. Unlike purely segmentation-focused approaches, it translates image-derived outputs into clinically interpretable geometric parameters and integrates them into a visible, repeatable CQS; however, the composite score still requires prospective validation against summative educational grades and clinical outcomes before high-stakes use.

### 4.1 Validation context and comparison with existing benchmarks

The present study includes external expert validation of the automated component measurements. It provides direct evidence that the automated geometric and depth measurements are closely aligned with independent expert assessment. A relevant benchmark is the 2024 study by Qutieshat et al., which compared algorithm-based 3D cavity grading with experienced academic-panel assessment and reported high sensitivity (0.98), moderate specificity (0.55), strong agreement with panel scoring (ICC3k = 0.857), and positive rank correlations (Spearman’s rho = 0.73; Kendall’s tau = 0.56; Qutieshat et al., 2024). The present framework complements that evidence by demonstrating strong expert agreement for 2D-derived component measurements and optional STL-derived depth. The final summative CQS was also validated against educational grade assessments done by three Independent Experts.

One of the primary benefits of the system is its uniform preprocessing and geometric normalisation, which reduces non-anatomical variability caused by inconsistent image acquisition settings. Orientation alignment, uniform scaling, automatic tooth localisation, and background removal using PCA produced geometrically consistent inputs from a range of image sources. PCA-based normalisation is a dependable technique for reducing pose variability while preserving inherent form features (Jolliffe and Cadima, 2016). Vertical alignment along the cavity’s main axis made measurements of isthmus width and intercuspal distance, which are otherwise very sensitive to camera angulation and tooth location, physically equivalent (Umbaugh, 2023).

Cavity and cusp segmentation was achieved using enhanced U-Net designs, demonstrating consistent performance despite variations in cavity geometry and imaging parameters. The strong segmentation results are consistent with the demonstrated effectiveness of U-Net models in biological image processing (Ronneberger et al., 2015). Composite loss functions boosted robustness in unbalanced datasets (Sudre et al., 2017), while data augmentation strategies enhanced model generalisation (Shorten and Khoshgoftaar, 2019; Perez and Wang, 2017). Accurate cusp segmentation supported generalizability across posterior teeth and benefited from residual feature-learning principles demonstrated in similar imaging tasks, enabling reproducible intercuspal distance calculation without explicit tooth-type labelling (Lim et al., 2017).

A central feature of this system is the measurement of global cavity shape similarity using EFDs, which provide translation-, scale-, and rotation-invariant contour representations and have long been validated for anatomical shape analysis (Falcão et al., 2017; Thapa et al., 2025). The external expert-validation data showed close agreement between automated EFD similarity and expert reference-match percentages (MAE = 1.32 percentage points; Pearson *r* = 0.981). The relatively high CQS weighting of EFD similarity reflects the clinical and educational importance of overall cavity outline conformance. Nevertheless, this weighting is a transparent expert-informed default rather than a statistically optimized coefficient. Future studies should estimate CQS weights using larger expert-panel outcomes, cross-validation, and calibration analyses to determine whether the proposed weight distribution best predicts faculty grading or restorative quality.

To capture functionally significant aspects of cavity design beyond global shape, the technique employs proportional geometric measurements, including intercuspal distance and normalised isthmus width. Skeleton-based isthmus identification and automated cusp centre detection made use of medial-axis-based algorithms (Falcão et al., 2017; Biasotti et al., 2006) and traditional morphological image analysis techniques (Lotufo et al., 2023; Telea and Jalba, 2012), with shortest-path optimisation based on graph theory (Mnih et al., 2015). These measures show that significant biomechanical assessment can be performed without requiring 3D imaging, as they were obtained solely from 2D segmentation masks.

The optional addition of STL-derived depth analysis broadens the framework’s analytical scope. By projecting 3D mesh vertices into 2D image space, segmentation masks and surface geometry could be connected (Sullivan and Kaszynski, 2019; Duran-Limon et al., 2026; Keleş et al., 2021). Regional depth aggregation and smoothness metrics provide clinically interpretable indicators of preparation depth and regularity, while best-fit plane references are consistent with point-cloud surface analysis methods (Li et al., 2020). However, the 3D component depends on accurate scaling, mesh quality, and image-to-mesh registration; these factors should be reported and externally tested in future work following AI imaging reporting standards (Tejani et al., 2024; American Dental Association, 2024).

### 4.2 Clinical and educational implications

The ability to assess cavity preparation quality using standard 2D images has potential translational value for formative dental education, remote review, and structured feedback in preclinical laboratories. The framework could help faculty calibrate assessments by making the features underlying a score visible: cavity outline conformity, intercuspal distance, isthmus width, proportional geometry, and optional depth/smoothness. The external expert validation strengthens the component-level evidence for this use case, because automated measurements showed close agreement with the mean of three external experts across the evaluated outputs. Nevertheless, the most appropriate near-term role remains human-supervised feedback, where the algorithm highlights measurable deviations and instructors retain final responsibility for educational and clinical interpretation. This human-in-the-loop position is consistent with current guidance for early-stage AI decision-support systems (American Dental Association, 2024; Vasey et al., 2022).

The study contains several limitations. The dataset size was relatively limited and derived from a restricted data source, which means that the robustness, stability, and generalizability of the proposed model across broader clinical settings. The current cusp segmentation model also does not yet account for all cusp configurations, such as mesiobuccal-mesiolingual linkages, which may enhance intercuspal analysis. In addition, 3D depth estimation depends on reliable STL scaling and accurate registration between 2D masks and 3D mesh coordinates.

In dental educational institutions, this system could assess plastic-teeth cavity preparations using standard 2D photographs already captured in clinics and preclinical laboratories. It generates a CQS from teachable features, including cavity outline conformity, intercuspal distance, isthmus width, and proportional geometry, and can also include depth and floor smoothness when 3D data are available. The external expert-validation data support the reliability of these component measurements and strengthen the translational framing of the system as a structured feedback and faculty-calibration tool. It can show students which geometric component reduced the score, help instructors align grading expectations, and support longitudinal monitoring of student progress. This analysis supports the preliminary validity of the CQS as an interpretable summary score for structured formative assessment, while further validation in larger and multi-institutional datasets remains necessary before high-stakes implementation. Recent reviews of AI in dental education identify procedural assessment and real-time feedback as underdeveloped areas, supporting the relevance of this direction while also emphasizing the need for standard frameworks, ethical oversight, and clinician-led validation (Ali et al., 2025).

## 5 Conclusion

The present study demonstrates a hybrid analytical paradigm in dental informatics. Deep learning provides reproducible segmentation of cavity and cusp regions, while conventional geometric and morphological analysis converts segmentation outputs into interpretable measures relevant to cavity preparation quality. By incorporating shape analysis, proportional geometry, optional STL-derived depth information, and external expert validation of component measurements into the CQS framework, the study proposes a transparent approach for objective, image-based cavity assessment. The evidence supports technical feasibility and preliminary external expert agreement for the evaluated component outputs. Our future work will include external multi-institutional testing, prospective educational implementation, and human-factors evaluation before the system is used for high-stakes assessment or clinical decision support.

## Data Availability

The raw data supporting the conclusions of this article will be made available by the authors, without undue reservation.

## Glossary

2D: Two-dimensional
3D: Three-dimensional
AdamW: Adam optimizer with decoupled weight decay
AI: Artificial intelligence
BCE: Binary cross-entropy
CAD/CAM: Computer-aided design/computer-aided manufacturing
CI: Confidence interval
CNN(s): Convolutional neural network(s)
CQS: Cavity Quality Score
CSV: Comma-separated values
CVAT: Computer Vision Annotation Tool
DSC: Dice Similarity Coefficient
EFD(s): Elliptic Fourier Descriptor(s)
HTML: HyperText Markup Language
ICC: Intraclass correlation coefficient
ICC(A,3): Absolute-agreement, average-measures ICC for three raters
ICC3k: Average-measures ICC notation used in the cited benchmark
ID: Identifier
IoU: Intersection over Union
MAE: Mean absolute error
MAPE: Mean absolute percentage error
NA: Not applicable
ORCID: Open Researcher and Contributor ID
PCA: Principal component analysis
ReLU: Rectified linear unit
RGB: Red, green, and blue
SD: Standard deviation
STL: Stereolithography/Standard Tessellation Language file format
U-Net: U-shaped convolutional neural-network architecture
