# Supplementary material for: Automated evaluation of dental cavity preparation quality using deep learning and anatomically informed geometric analysis
Source: Front Artif Intell. 2026 Aug 11;9:1884843. doi: 10.3389/frai.2026.1884843 (PMC13503532; doi:10.3389/frai.2026.1884843)
Supplement: Supplementary file 1 [file supplementary_file_1.zip › Molar Cavity Paper supported data/Expert Validation/ICC Table for Experts.docx]

| **Expert-rated domain** | **n** | **ICC(A,3)** | **95% CI** |
| --- | --- | --- | --- |
| EFD reference-match percentage | 25 | 0.947 | 0.873-0.973 |
| Isthmus width, pooled isthmus 1 and 2 | 40 | 0.989 | 0.977-0.993 |
| Cusp-pair distance, pooled pair 1 and 2 | 48 | 0.994 | 0.990-0.996 |
| Depth regional maxima, pooled regions | 6 | 0.995 | 0.969-0.999 |
| Final depth D by case | 2 | 0.995 | Not estimable because n=2 |
