# Supplementary material for: Automated evaluation of dental cavity preparation quality using deep learning and anatomically informed geometric analysis
Source: Front Artif Intell. 2026 Aug 11;9:1884843. doi: 10.3389/frai.2026.1884843 (PMC13503532; doi:10.3389/frai.2026.1884843)
Supplement: Supplementary file 1 [file supplementary_file_1.zip › Molar Cavity Paper supported data/Figure 1s.docx]

*Figure 1.	Standardisation of two-dimensional molar photographs and segmentation masks through spatial scaling and principal-axis anatomical alignment.*
